# MULTIMODAL AFFECTIVE ANALYSIS OF FACIAL AND VOCAL EXPRESSIVITY USING SMARTPHONE AND DEEP LEARNING ANALYSIS

**DOI:** 10.1093/geroni/igac059.2221

**Published:** 2022-12-20

**Authors:** Heejung Kim, Youngshin Cho, Sunghee Lee, Chaehyeon Kang

**Affiliations:** Yonsei University, Seoul, Seoul-t'ukpyolsi, Republic of Korea; Yonsei University, Seoul, Seoul-t'ukpyolsi, Republic of Korea; BRFrame, Seoul, Seoul-t'ukpyolsi, Republic of Korea; Yonsei University, Seoul, Seoul-t'ukpyolsi, Republic of Korea

## Abstract

Limited expressivity of emotion is one of the most common symptoms of major depression, particularly in older adults. Although assessing facial and vocal expressivity is very important for accurate clinical evaluation of geriatric depression, research has rarely examined older adults via telehealth technology. This study aims to quantify facial and vocal expressivity via a multimodal affective system with deep learning. A total of 19 Korean adults aged over 65 years with severe depressive symptoms participated in this research. Using smartphone video recording, 1,429 facial and vocal data were collected between July and December 2020. Recorded videos were transmitted automatically to the cloud system. Basic facial movements were extracted using combined video frames and mel spectrogram images. Compared to the AI hub of Korean images from big data, mood status was classified into seven categories (anger, disgust, fear, happiness, neutrality, sadness, and surprise). Frequencies of each mood were coded into continuous variables for each participant in each recording. When comparing video and text prediction to determine “true labels,” the overall accuracy was 0.69, with F1 scores ranging from 0.57 to 0.79. In addition, the most common emotions were angry, happy, neutral, sad, and surprised. This study suggests that smartphone-recorded video could function as a useful tool for quantifying mood expressivity. This study established a preliminary method of affective assessment for older adults for telecare use based on socially assistive technology at a distance from the clinic.

